# Metabolic changes and inflammation in cultured astrocytes from the 5xFAD mouse model of Alzheimer’s disease: Alleviation by pantethine

**DOI:** 10.1371/journal.pone.0175369

**Published:** 2017-04-14

**Authors:** Manuel van Gijsel-Bonnello, Kévin Baranger, Philippe Benech, Santiago Rivera, Michel Khrestchatisky, Max de Reggi, Bouchra Gharib

**Affiliations:** Aix Marseille Univ, CNRS, NICN, Marseille, France; Torrey Pines Institute for Molecular Studies, UNITED STATES

## Abstract

Astrocytes play critical roles in central nervous system homeostasis and support of neuronal function. A better knowledge of their response may both help understand the pathophysiology of Alzheimer’s disease (AD) and implement new therapeutic strategies. We used the 5xFAD transgenic mouse model of AD (Tg thereafter) to generate astrocyte cultures and investigate the impact of the genotype on metabolic changes and astrocytes activation. Metabolomic analysis showed that Tg astrocytes exhibited changes in the glycolytic pathway and tricarboxylic acid (TCA) cycle, compared to wild type (WT) cells. Tg astrocytes displayed also a prominent basal inflammatory status, with accentuated reactivity and increased expression of the inflammatory cytokine interleukin-1 beta (IL-1β). Compensatory mechanisms were activated in Tg astrocytes, including: i) the hexose monophosphate shunt with the consequent production of reducing species; ii) the induction of hypoxia inducible factor-1 alpha (HIF-1α), known to protect against amyloid-β (Aβ) toxicity. Such events were associated with the expression by Tg astrocytes of human isoforms of both amyloid precursor protein (*APP*) and presenilin-1 (*PS1*). Similar metabolic and inflammatory changes were induced in WT astrocytes by exogenous Aβ peptide. Pantethine, the vitamin B5 precursor, known to be neuroprotective and anti-inflammatory, alleviated the pathological pattern in Tg astrocytes as well as WT astrocytes treated with Aß. In conclusion, our data enlighten the dual pathogenic/protective role of astrocytes in AD pathology and the potential protective role of pantethine.

## Introduction

Astrocytes play a central role in brain metabolism for neuronal support. In response to pathological situations, they become reactive and display distinct phenotypes [1]. In AD, activated astrocytes display ambivalent functions since they contribute to Aβ production [2], but they also limit plaque growth [3]. Most *in cellulo* studies addressing the role of astrocytes in AD have focalized on the effects of exogenous Aβ on WT cells. Only few studies have addressed this question on primary astrocyte cultures from transgenic AD mice. In the Tg2576 model, pro-inflammatory cytokines and Aβ42 oligomers and fibrils, increased the levels of β-secretase BACE1, APP, as well as β-secretase processing of APP that can lead to increased Aβ production [2]. In the same model, IFN-γ stimulated BACE1 expression and APP processing by this secretase [4]. Astrocytes from double-transgenic APPswePS1dE9 mice showed a “chronic” activation that coincides with impairment of normal astrocyte function [5], while 5xFAD astrocytes showed reduced Aβ internalization [6]. In the presenilin 1 (PS1) knock-in (PS1_M146V_) mouse model, cultured astrocytes showed an enhanced ceramide-induced apoptosis, in contrast to WTs [7].

The development of AD pathogenesis in human as in animal models could be linked to metabolic disorders, including alterations in bioenergetic pathways and TCA cycle [8,9]. Energetic and mitochondrial dysfunction may contribute to neuroinflammation, which in turn could cause mitochondrial dysfunction [10]. Neuroinflammation can be revealed in isolated glia from AD mice [5,11]. Neuroinflammation in AD involves mediators such as IL-1β, IL-6, TNF-α and TGF-β that are upregulated in patients compared to healthy individuals [12–15], as well as in the 5xFAD transgenic mouse model of AD (Tg) [16,17]. The latter recapitulates major features of AD pathology, including exacerbated Aβ deposition, accumulation of its immediate precursor, the neurotoxic APP C-terminal fragment C99, and gliosis. All these events are detectable in 5xFAD mice at early phases of the pathology, between 2 and 4 months [16–20]. Aβ and C99 accumulation stimulates pathogenesis and neuroinflammation in AD mice [21,22]. Aß promotes IL-1β production and secretion by cultured astrocytes [23,24], which in turn stimulates Aβ production and consequent neuronal demise [25]. For these reasons, inflammation is considered to aggravate AD pathogenesis.

In an attempt to explore early pathogenic events in AD that could be mediated by astrocytes, we used primary astrocyte cultures generated from newborn 5xFAD and WT mice. We explored the alterations in energy metabolism and the inflammatory response of these astrocytes, with or without exposure to oligomeric Aβ (oAβ). The latter is known to stimulate astrocyte reactivity and initiate neuronal damage [26,27]. We determined the subsequent alterations in the glycolytic pathway and TCA cycle, as well as proteasome activity and the levels of HIF-1α, which has been reported to be neuroprotective in AD [28] and to reduce astrocyte activation after Aβ treatment [29,30].

Because pantethine has anti-inflammatory properties, astrocytes were pre-treated with this compound to determine its potential protective effects in AD. Pantethine is a dietary low-molecular-weight thiol, precursor of vitamin B5, known to reduce metabolic dysfunctions [31–34], decrease inflammation and mediate immune responses [35,36]. In our study we showed that pantethine moderated metabolic dysfunctions and astrocyte reactivity linked to AD-like pathogenesis, suggesting that the compound could help maintain homeostasis and function of the AD brain.

In summary, our study provides further insight into the pathophysiological response of astrocytes, which could improve the knowledge of AD pathogenesis and pave the way for new anti- neurodegenerative therapeutic strategies that target astrocyte-mediated processes.

## Materials and methods

### 5xFAD primary astrocyte cultures

5xFAD (Tg) mice express five familial AD mutations in the amyloid precursor protein (APP) [20] and presenilin1 (PS1) genes, under the control of the neuron-specific Thy1 promoter. Primary astrocyte cultures were prepared from the neocortices of 1- to 3-day-old Tg and WT littermate control mice as described previously [37,38]. All experimental procedures, including the efforts to alleviate animal suffering, were approved by the ethics committee of the Faculty of Medicine (Aix-Marseille University) in accordance with National and European regulations (EU directive N° 2010/63), and in agreement with the authorization for animal experimentation attributed to the laboratory by the Prefecture des Bouches-du-Rhône (permit number: D 13 055 08). All efforts were made to minimize animal suffering and reduce the number of animals used.

Gestational mice were anesthetized with pentobarbital (0.36 g/Kg) and the fetuses were obtained by cesarean section and then decapitated. After removal of the meninges, half of Tg and WT brains were dissociated into a single-cell suspension by trypsinization and mechanical disruption. The cells were seeded at an average density of 10^5^ cells/cm^2^ in flasks or in 24-well plates on coverslips and grown at 37°C in a 5% CO_2_ humidified atmosphere in Dulbecco’s modified Eagle medium (DMEM) supplemented with 10% FBS, 100 units/mL penicillin/streptomycin solution (all from Thermo Fisher Scientific, Saint-Aubin, France). The culture medium was renewed 3–5 days after seeding and subsequently twice a week. After 3 weeks, the cultures were shaken at 250 rpm for 30 min on a horizontal shaker to remove microglial cells. These culture conditions yielded >85% of immunopositive cells stained with monoclonal anti-glial fibrillary acidic protein (GFAP, 1/300, Millipore, Molsheim, France) and less than 15% of immunopositive microglial cells stained with the anti-Iba1 polyclonal antibody (1/300, Wako, Sobioda, Montbonnot, France). Goat anti-mouse Alexa Fluor^®^ 488 and goat anti-rabbit Alexa Fluor^®^ 568 secondary antibodies were used respectively (ThermoFisher Scientific). Two days before the experiments, confluent 3-week *in vitro* cultures were maintained in serum free Neurobasal medium (ThermoFisher Scientific) supplemented with 2% of B27 and 100 units/mL of penicillin/streptomycin. The other half of the brain was quick-frozen after removal and used for biochemical analyses.

### Aβ_1–42_ preparation

Human synthetic Aβ_1–42_ in hexafluoroisopropanol (HFIP) and reverse sequence Aß peptide (rAβ) were purchased from (Bachem, Bubendorf, Switzerland). Oligomeric species were prepared as described previously [39,40]. Briefly, HFIP was removed under vacuum in a Speed Vac, the peptide film was resuspended to 0.2 mM in anhydrous dimethyl sulfoxide (DMSO; Sigma-Aldrich, Saint-Quentin-Fallavier, France), bath-sonicated for 10 min, then further diluted to 100 μM in ice cold phenol red free F-12 media and incubated at 4°C for 24 h before dilution to final concentration or kept at -80°C. rAβ was prepared as Aβ_1–42_. Oligomeric Aβ (oAβ) formation was controlled by western blots probed with the 6E10 monoclonal antibody (Covance, Ozyme, Saint-Quentin en Yvelines, France). Cells were incubated with oAβ_1–42_ at 1 μM for 6 and 24 h unless otherwise indicated in the text.

### Pantethine treatment

Pantethine for cell cultures was purchased from Sigma-Aldrich. Purity of the preparation was ascertained using high performance liquid chromatography-coupled mass spectrometry (HPLC-LC-UV-MSD). Pantethine was added to astrocyte cultures at the concentration of 1 mM for 18 h. Previous experiments on primary human cells have shown that maximal cellular effects were obtained under these conditions without side effects [41]. Astrocytes were then washed in PBS and used in the assays.

### Metabolomics

Metabolic analysis in Tg and WT astrocytes, treated or not with pantethine, was carried out by IC-MS/MS quantitative analysis using ICS-5000+Ion Chromatograph and 4000 Qtrap Mass Spectrometer, with a method adapted by Metatoul (LISBP-INSA, Toulouse, France). Experiments were performed on astrocytes seeded at 3x10^6^ cells in T25 flasks. At the end of the incubation, cells were washed with cold PBS and frozen in liquid nitrogen. For extract preparation, a mixture of ACN/MeOH/H20 (4:4:2 v/v) was added with an internal standard (IDMS). Cells were then scraped off the flask, the solvent was removed under vacuum and the samples were stored at -80°C pending analysis.

### Enzymatic activities

#### Glycolysis

Astrocyte cultures from Tg and WT pups were treated or not with pantethine. They were then incubated or not with 1 μM oAβ for 24 h and washed twice with 20 mM potassium phosphate buffer (pH 7). Cells were scraped off the flask, lysed in the appropriate buffer by sonication and protein content and enzymatic activities determined. Glucose-6-phosphate dehydrogenase (G6PD) activity was measured as previously described [42] by measuring the rate of absorbance increase at 340 nm from the conversion of NADP^+^ to NADPH by G6PD. Substrate concentrations used were glucose-6-phosphate (G6P, 200 μM) and NADP^+^ (100 μM). NADPH was measured as described [43]. Pyruvate Kinase (PK) activity was measured by a coupled enzymatic assay NADH/lactate dehydrogenase (LDH) [44]. Decrease in OD_340nm_ due to NADH oxidation was monitored using a UV-DU 800 spectrophotometer (Beckman Coulter, Villepinte, France).

#### TCA cycle

Mitochondria purification from astrocytes was performed as described previously [45]. Tg and WT astrocytes treated or not with pantethine and/or 1μM oAβ for 24 h were collected by trypsinization. Samples were centrifuged at 200 x *g* for 10 min and washed in PBS. The supernatants were discarded and the pellets were incubated in hypotonic buffer (0.15 mM MgCl_2_, 10 mM KCl, 10 mM Tris-HCl, pH7.6) at 4°C for 5 min. After the addition of an equal volume of homogenization buffer (0.15 mM MgCl_2_, 10 mM KCl, 10 mM Tris-HCl, 0.4 mM phenylmethylsulfonyl fluoride, 250 mM saccharose, pH 7.6) twice concentrated, samples were manually homogenized with a Dounce Potter homogenizer. Cell extracts were centrifuged at 900 x *g* for 10 min, followed by supernatant centrifugation at (10,000 x *g* for 10 min). Mitochondrial pellets were resuspended and the total amount of protein was quantified using Bio-Rad *DC*^™^ protein assay kit following manufacturer’s instructions (Bio-Rad, Marne-La-Coquette, France). All steps were carried out at 4°C.

α-ketoglutarate dehydrogenase complex (α-KGDHc) activity was determined as described previously [46]. Briefly, in a pH 7.4 buffer containing 35 mM KH_2_PO_4_, 5 mM MgCl_2_, 0.5 mM EDTA, 0.5 mM NAD, 0.2 mM thiamine pyrophosphate, 0.04 mM CoA-SH, and 2 mM α-ketoglutarate. α-KGDHc activity was determined as NAD reduction assayed at 340 nm (ε = 6220 M^-1^cm^-1^) using a UV-DU 800 spectrophotometer (Beckman Coulter).

Succinate dehydrogenase (SDH) activity was determined spectrophotometrically using 2,6- dichlorophenolindolphenol (DCPIP) as an artificial electron acceptor and succinate as the substrate [47]. The assay was performed at room temperature in an assay buffer composed of 25 mM KHPO_4_ pH 7.4, 20 mM succinate, 50 μM decylubiquinone, 5 μM rotenone, 2 μM antimycin A and 10 mM NaN_3_. Following 15 min incubation at room temperature, the baseline absorbance at 600 nm was recorded and the reaction was initiated by adding 50 μM DCPIP. SDH activity was calculated using the molar absorption coefficient of reduced DCPIP (ε = 21 mM^-1^.cm^-1^).

### Immunocytochemistry

Tg and WT astrocytes were cultured on glass coverslips, pre-treated or not with pantethine (1 mM), washed and exposed or not to 1 μM of oAβ for 24 h. Cells were then washed and fixed with 4% paraformaldehyde and stained with a polyclonal rabbit anti-GFAP (1/300; Dakocytomation, France) and labeled with a secondary goat anti-rabbit Alexa Fluor^®^-488-conjugated antibody (1/500, Thermo Fisher Scientific). Activation was quantified using a confocal microscope LSM-700 equipped with Zen software (Zeiss, Jena, Germany). The number of stellate cells characterized by cellular hypertrophy and increased staining of GFAP was determined in 10 randomly selected 10^5^ μm^2^ non-overlapping fields from all the pictures obtained. Activated cells were expressed as the percentage of the total number of cells in the field.

### Cell viability

Cell viability was measured by the methylthiazolyldiphenyl-tetrazolium bromide (MTT; Sigma-Aldrich) reduction assay [48]. At the end of treatment with pantethine and/or oAβ, the medium was replaced by DMEM medium containing 0.1 mg/mL of MTT for 3 h at 37°C. Lastly, the amount of reduced MTT (formazan) solubilized in dimethylformamide (DMF) was determined as OD_560nm_ using DU 800 spectrophotometer (Beckman Coulter).

### IL-1β expression

Murine *IL-1β*, human *APP* and *PS1* gene expression in astrocyte cultures and brain was determined using quantitative PCR. Total RNA was extracted from cortex and astrocyte samples using the Nucleospin RNA II kit (Macherey-Nagel, Hoerd, France) according to the manufacturer’s instructions. The cDNA was synthesized from 500 ng of RNA using random primers (ThermoFisher Scientific) and Molony Murine Leukemia Virus Reverse Transcriptase (M-MLV RT, ThermoFisher Scientific). Quantitative PCR was performed with an Applied Biosystems 7500 Real-Time PCR System (Applied Biosystems, ThermoFisher Scientific) using TaqMan probes to detect the amplification products. For each experiment, three cDNA samples from independent cultures were analyzed in duplicate with the following probes *IL-1β* (Mm 01336189_m1), *APP* (Hs 00169098_m1), *PS1* (Hs 00997789_m1) and *Gapdh* (Mm 99999915_g1) as calibrator.

### ELISA assays

The levels of IL-1β in astrocyte culture supernatants and in cortex extracts were analyzed by ELISA assay (Peprotech, Neuilly-sur-Seine, France) and RayBio (Tebu-bio, Le Perray-en-Yvelines, France) according to the manufacturer’s instructions. For detection of Aβ1–40 and Aβ1–42 levels in astrocyte culture supernatants, ELISA kit (Life Technologies) was used according to the manufacturer’s recommendations.

### Western blot analysis

Whole astrocyte lysates were prepared by incubating cells on ice for 30 min in buffer containing 100 mM PBS, 2 mM NaF, 2.5 mM Na_4_P_2_O_7_, 1 mM Na_3_VO_4_, 1% Triton X-100, pH 7.2 supplemented with 1% protease inhibitor cocktail (Sigma-Aldrich). The frozen brains were homogenized in the same buffer and centrifuged at 12,000 x *g* for 20 min at 4°C. Protein concentration was quantified by using the Bio-Rad *DC*^™^ protein assay kit following the manufacturer’s instructions (Bio-Rad). Equal amounts of protein were separated on either 12% or 15% SDS-PAGE and transferred to nitrocellulose membranes (GE Healthcare, Dutscher, Brumath, France). After blocking, membranes were probed with the following antibodies: monoclonal anti-APP N-terminal (22C11, 1/500, Millipore, Molsheim, France), monoclonal anti-Aβ (6E10, 1/500, Covance), polyclonal anti-APP C-terminal (APP-CTF, 1/2000, Sigma-Aldrich), monoclonal anti-HIF-1α (1/500; R&D Systems Europe, Bio-Techne, Lille, France) and monoclonal anti-actin (1/5000, Sigma-Aldrich). Membranes were then incubated with the appropriate horseradish peroxidase-conjugated secondary IgG antibodies (Jackson Immunoresearch, West Grove, PA, USA) immunoblot signals were visualized with ECL chemiluminescence kit (GE Healthcare). Quantitative assessments were obtained by densitometric scanning using ImageJ software.

### Proteasome activity assay

Astrocytes were placed on ice and homogenized in extraction buffer (20 mM Tris-HCl pH 7.8, 1 mM DTT, 1 mM ATP, 10% glycerol, 0.5 mM EDTA, 0,5% Igepal, and 5 mM MgCl_2_) [49]. The lysates were centrifuged at 14,000 x *g* at 4°C for 15 min and the resulting supernatants were placed on ice and assayed for protein concentrations using the Bio-Rad *DC*^™^ kit. Chymotrypsin and trypsin proteasome activities were analyzed using fluorogenic substrates: Suc-Leu-Leu-Val-Tyr-7-amino-4-methyl coumarin (Millipore) and Z-Ala-Arg-7-amino-4-methyl coumarin (Bachem). Samples containing 20 μg of protein were incubated with fluorogenic substrate (final concentration, 50 μM) in a buffer containing 20 mM Tris-HCl, pH 7.8, 5 mM MgCl_2_, 1 mM DTT, and 1 mM ATP. All assays were done in triplicate. Samples were incubated at 37°C for 60 min in a 96-well plate. Fluorescence was determined at 355 nm excitation/460 nm emission in a Microplate Spectrofluorometer SpectraMax M2 (Molecular Devices, Wokingham, UK) and proteolytic activities were calculated on the basis of total cellular protein. The specificity of the proteasome assay was confirmed using MG132 (0.5 μM), a selective proteasome inhibitor (Enzo Life Sciences, Villeurbanne, France).

### Statistical analysis

All experiments were performed at least three times and the data are shown as mean ± SD. Non-parametric Kruskall Wallis ANOVA was performed to determine statistical significance between experimental groups. A *p* value less than 0.05 was considered significant.

## Results

### Astrocyte metabotyping

Glycolysis coupled with TCA cycle constitutes the main pathway of energy generation through ATP synthesis. Accordingly, a global metabolomic profiling was performed on lysates from WT and Tg astrocytes. Table 1 shows a list of significant metabolites from several key biological pathways that characterize the transgenic-associated changes and the effects of pantethine treatment.

**Table 1:** Relative abundance of distinct metabolites in Tg and WT astrocytes, treated or not with pantethine. Intracellular levels of different metabolites were determined. The table shows normalized data expressed relative to the abundance of the untreated WT group and are shown as the mean values ± SD from three independent experiments (n = 3 per group); (*, values significantly different between Tg and WT astrocytes; #, values significantly different compared to the corresponding untreated groups; *p*≤0.05).

Cell type	WT	WT	Tg	Tg
Pantethine	−	+	−	−
Glycolytic pathway				
PEP	1±0.2	1.07±0.19	1.39±0.15*	1.43±0.13
G6-P	1±0.19	0.95±0.17	0.63±0.08*	0.54±0.05
Sed7P	1±0.15	0.91±0.19	0.47±0.09*	0.64±0.08^#^
Gly-3P	1±0.19	0.97±0.16	0.37±0.1*	0.53±0.08^#^
TCA cycle metabolites				
α-KG	1±0.12	0.94±0.13	1.25±0.15*	0.56±0.11^#^
Succinate	1±0.13	0.99±0.12	0.92±0.11	0.72±0.09^#^
Fumarate	1±0.21	0.94±0.19	0.65±0.09*	0.99±0.13^#^
Cis-aconitate	1±0.25	0.94±0.18	1.37±0.3	1.29±0.28
Citrate	1±0.25	0.86±0.21	1.24±0.29	1.26±0.22
Nucleotides				
ATP	1±0.08	0.97±0.12	1.01±0.13	1.44±0.14^#^
AMP	1±0.07	0.90±0.19	0.80±0.11*	1.07±0.15
CMP	1±0.15	0.96±0.19	0.60±0.11*	1.00±0.22^#^
CTP	1±0.12	0.98±0.15	1.03±0.11	1.16±0.13
GDP	1±0.15	0.95±0.13	0.91±0.11	1.03±0.16
ADP	1±0.18	0.98±0.16	1.10±0.12	1.19±0.16

In the glycolytic pathway, Tg astrocytes exhibited contrasting changes compared to WT, such as an elevation of 39% of phosphoenoylpyruvate (PEP) abundance. PEP is substrate of pyruvate kinase to generate pyruvate and one molecule of ATP. Levels of metabolites involved in the alternative pathways, namely glucose-6-phosphate (G6-P), sedoheptulose-7-phosphate (Sed7P) and glycerol-3-phosphate (Gly-3P) dropped by 37%, 53% and 63%, respectively, compared to WT. In the TCA cycle, levels of α-ketoglutarate (α-KG), a substrate of the mitochondrial enzyme α-ketoglutarate dehydrogenase (α-KGDHc), increased by 25% while levels of succinate, the product of this enzymatic reaction, remained unchanged. Succinate is further metabolized into fumarate by succinate dehydrogenase (SDH). Fumarate levels were decreased by 35% in Tg astrocytes compared to WT. Levels of cis-aconitate and citrate were unchanged between WT and Tg astrocytes. All these results suggested significant defects in the glycolytic pathway and TCA cycle in Tg compared to WT astrocytes.

Pantethine treatment had no effect on these parameters in WT astrocytes, while in Tg, it mitigated the unbalanced production of metabolites involved in the energetic alternative pathway. Indeed, Sed7P and Gly-3P levels increased by 36% and 43% respectively, compared to untreated astrocytes. Concerning the TCA cycle, pantethine treatment decreased α-KG and succinate levels by 55% and 22%, respectively, while it increased fumarate levels by 34% compared to untreated cells. These results underlined that pantethine corrected and stabilized the levels of energetic metabolites possibly to maintain the cellular energetic balance. As a consequence, the levels of nucleotides (*i*.*e*., AMP and CMP) that are essential cofactors in cell homeostasis, decreased by 20% and 40% in Tg astrocytes compared to WT, a decrease that was prevented by pantethine treatment. Importantly, ATP had its levels increased by 44% in pantethine-treated Tg astrocytes compared to untreated. Changes in the levels of this metabolite should be considered in association with changes in the enzymatic activities of key enzymes of the glycolysis pathway and TCA cycle: glucose-6-phosphate dehydrogenase (G6PD), pyruvate kinase (PK), α-ketoglutarate dehydrogenase (α-KGDHc) and succinate dehydrogenase (SDH).

### Enzymatic activities

**Fig 1:**
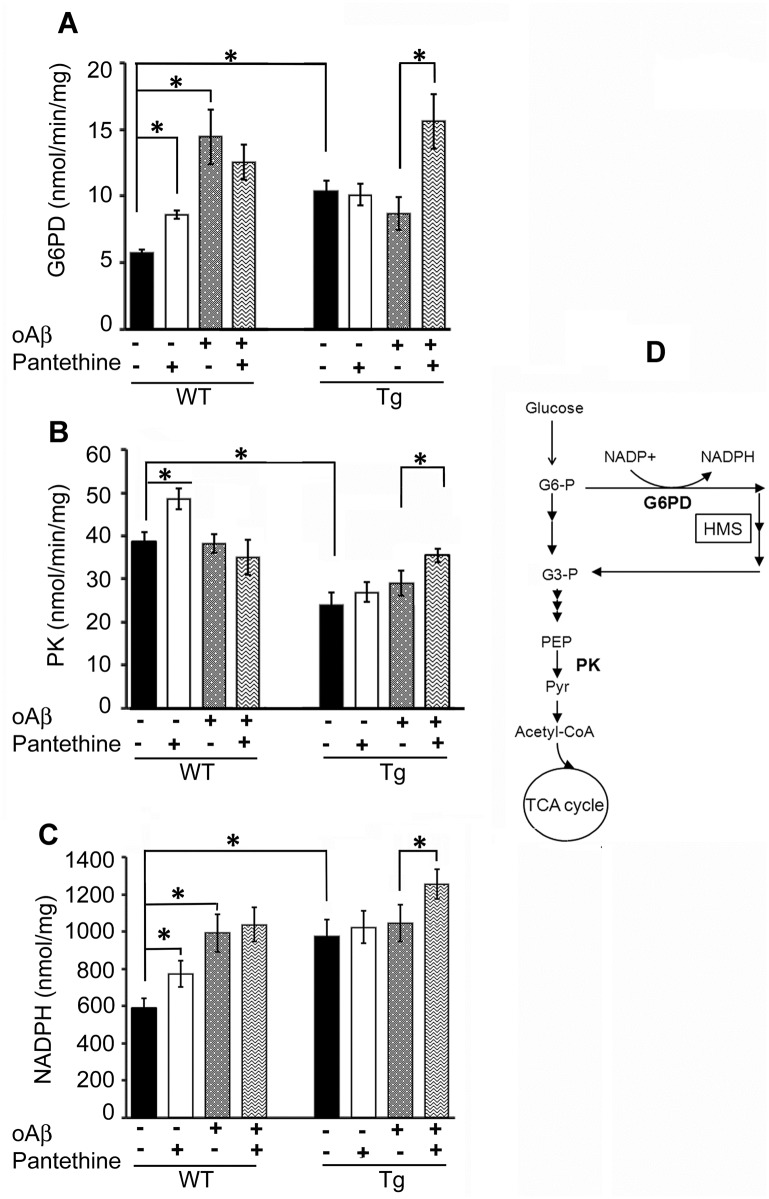
Enzymatic activities involved in the glycolytic pathway. Tg and WT astrocytes were treated or not with pantethine and then exposed or not to exogenous oligomeric Aβ (oAβ). Enzymatic activity and NADPH levels were determined on cell extracts. (A) Glucose-6-phosphate dehydrogenase (G6PD) activity. (B) Pyruvate kinase (PK) activity. (C) NADPH levels. Results are the mean values ± SD from three independent experiments (n = 3 per group). Enzyme activities are expressed as nmol/min/mg protein and NADPH levels are expressed as nmol/mg protein (*, significant difference between experimental groups; *p*<0.05). (D) Schematic representation of glucose metabolism enlightening the hexose monophosphate shunt (HMS) and PK (G6-P, glucose-6P; G-3P, glyceraldehyde-3P; PEP, phosphoenoylpyruvate; pyr, pyruvate).

In the glycolytic pathway, G6PD and PK activities were investigated. G6PD is involved in the hexose monophosphate shunt (HMS), which yields NADPH. Pyruvate kinase (PK) catalyzes the ultimate step of the glycolytic pathway; it dephosphorylates phosphoenolpyruvate (PEP) to generate pyruvate (Pyr), the latter being the entry point into the TCA cycle. Fig 1A shows that the basal G6PD activity was significantly 2-fold higher in Tg astrocytes than in WT cells, while PK activity was significantly decreased by 37% (Fig 1B). Exposure of cells to oAβ induced a 3-fold increase of G6PD activity in WT astrocytes, whereas in Tg cells the activity, already high, remained unchanged.

Pantethine treatment increased both G6PD and PK activity in WT by about 40% and 25%, respectively. In Tg cells, the activities were increased by 80% and 22%, respectively, when pantethine treatment was combined to oAβ. G6P metabolism *via* the HMS yields NADPH; in agreement, astrocyte NADPH levels paralleled the changes of G6PD activity described above (Fig 1C).

**Fig 2:**
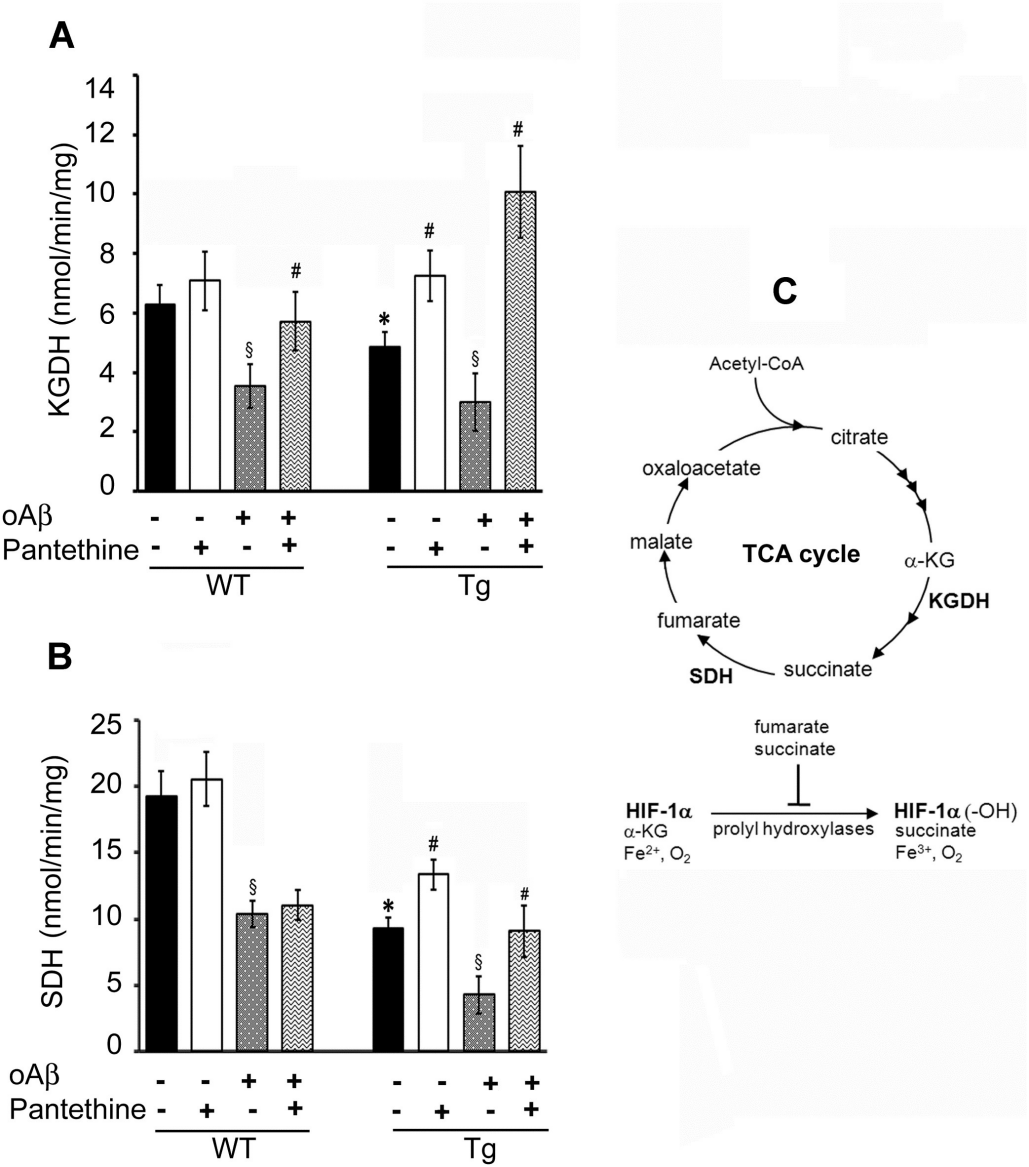
Enzymatic activities involved in the TCA cycle. Tg and WT astrocytes were treated or not with pantethine, then exposed or not to oAβ. Enzymatic activities were determined on mitochondrial preparations. (A) α-Ketoglutarate dehydrogenase (KGDH) activity. (B) succinate dehydrogenase (SDH) activity. Results are the mean values ± SD from three independent experiments (n = 3 per group) and are expressed as nmol/min/mg protein (*, significant difference with the WT group; §, significant difference with the corresponding group not exposed to oAβ; #, significant difference with the corresponding group untreated with pantethine; *p*<0.05). (C) Schematic representation of the TCA cycle enzymes and metabolites involved in HIF-1α hydroxylation (α-KG: α-ketoglutarate; HIF-1α: hypoxia inducible factor-1α; HIF-1α (-OH): hydroxylated HIF-1α).

To determine whether changes in the levels of TCA cycle metabolites matched changes in enzyme-related activities, we measured the specific activities of α-KGDHc and SDH (Fig 2). α-KGDHc activity leads to the production of succinate, which is used by SDH to produce fumarate. The activities of α-KGDHc and SDH were significantly reduced by 15% and 50% in Tg astrocytes, respectively, compared to WT. Accordingly, exposure to oAβ inhibited the two enzymes in both WT and Tg cells. As a consequence, the decrease of α-KGDHc activity resulted in an increase of its substrate α-KG. Moreover, the decrease of SDH activity might explain the decrease in the fumarate levels observed in Tg cells (Table 1).

Pantethine treatment restored at least in part the low enzymatic activities in Tg cells. The restoration of α-KGDH and SDH activities is likely to be responsible for the consumption of α-KG and succinate, respectively, as illustrated by the reduced levels of these substrates. The treatment restored the enzymatic activities in both cell types after addition of oAβ (with the exception of SDH activity in WT). Pantethine had no effect under physiological conditions, *i*.*e*., in WT astrocytes not exposed to oAβ.

### Transgenic astrocyte activation and inflammatory status

**Fig 3:**
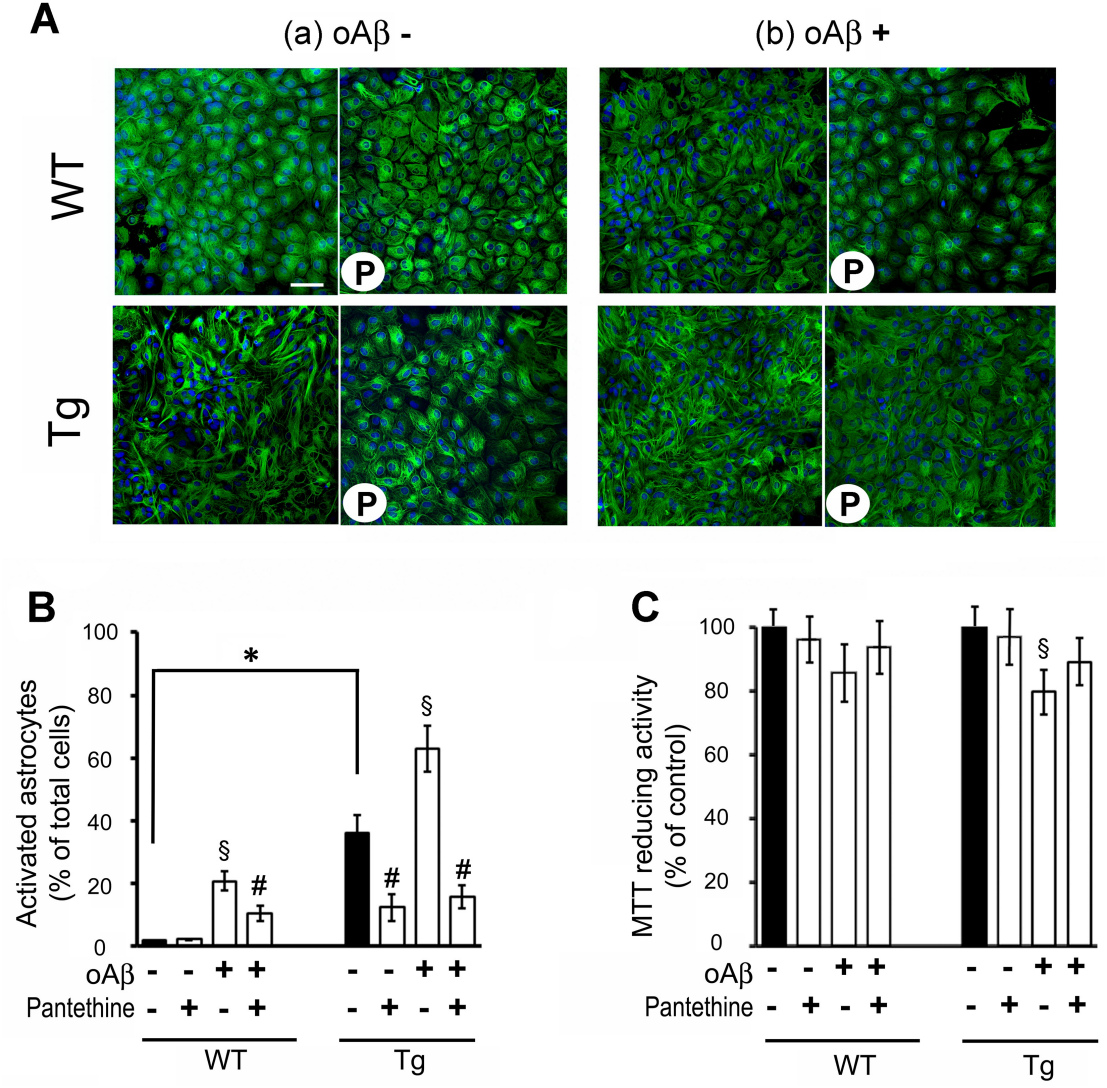
Tg and WT astrocyte activation status. Tg and WT astrocytes were treated or not with pantethine, then exposed or not to oAβ. (A) Confocal microscopy images of astrocyte morphology monitored by GFAP labeling (green); nuclei are labeled with Hoechst (blue). a, Quiescent astrocytes; b, astrocytes exposed to oAβ; P, pantethine treatment. Each picture is representative of images obtained from at least three independent cultures. (B) Astrocyte activation quantified by counting the number of activated cells (stellar shape) as a percent of the total number of cells in 10 randomly selected 10^5^ μm^2^ fields from all the pictures obtained. (C) Astrocyte viability determined using the MTT assay. The diagrams are mean values ± SD from three independent experiments (n = 3 per group) (*, significant difference between WT and Tg groups; §, significant difference with groups not exposed to oAβ; #, significant difference with the corresponding group untreated with pantethine; *p*<0.05). Scale bars: 100 μm.

Since metabolic disorders are associated with inflammation, we characterized the inflammatory status in Tg astrocytes relative to WT. Reactive astrocytes are characterized by a stellar shape and upregulation of glial fibrillary acidic protein (GFAP) levels. In our experimental conditions, about 40% of naïve Tg astrocytes displayed such a reactive phenotype, in contrast to only 2% in WT (Fig 3A and 3B). Following exposure to oAβ, the percentage of activated astrocytes significantly rose to about 60% in Tg and modestly to 20% in WT cells. Pantethine treatment significantly reduced astrocytes reactivity in all experimental conditions (Fig 3B). The MTT reduction assay revealed that exposure to drugs, either oAβ or pantethine, had no significant effect on WT astrocyte viability. However, we observed a slight but significant decrease of viability of Tg cells exposed to oAβ, which was prevented by pantethine (Fig 3C). As a control, reverse Aβ (rAβ) had no effect on astrocyte activation and enzyme activities described below (data not shown).

### IL-1β expression

**Fig 4:**
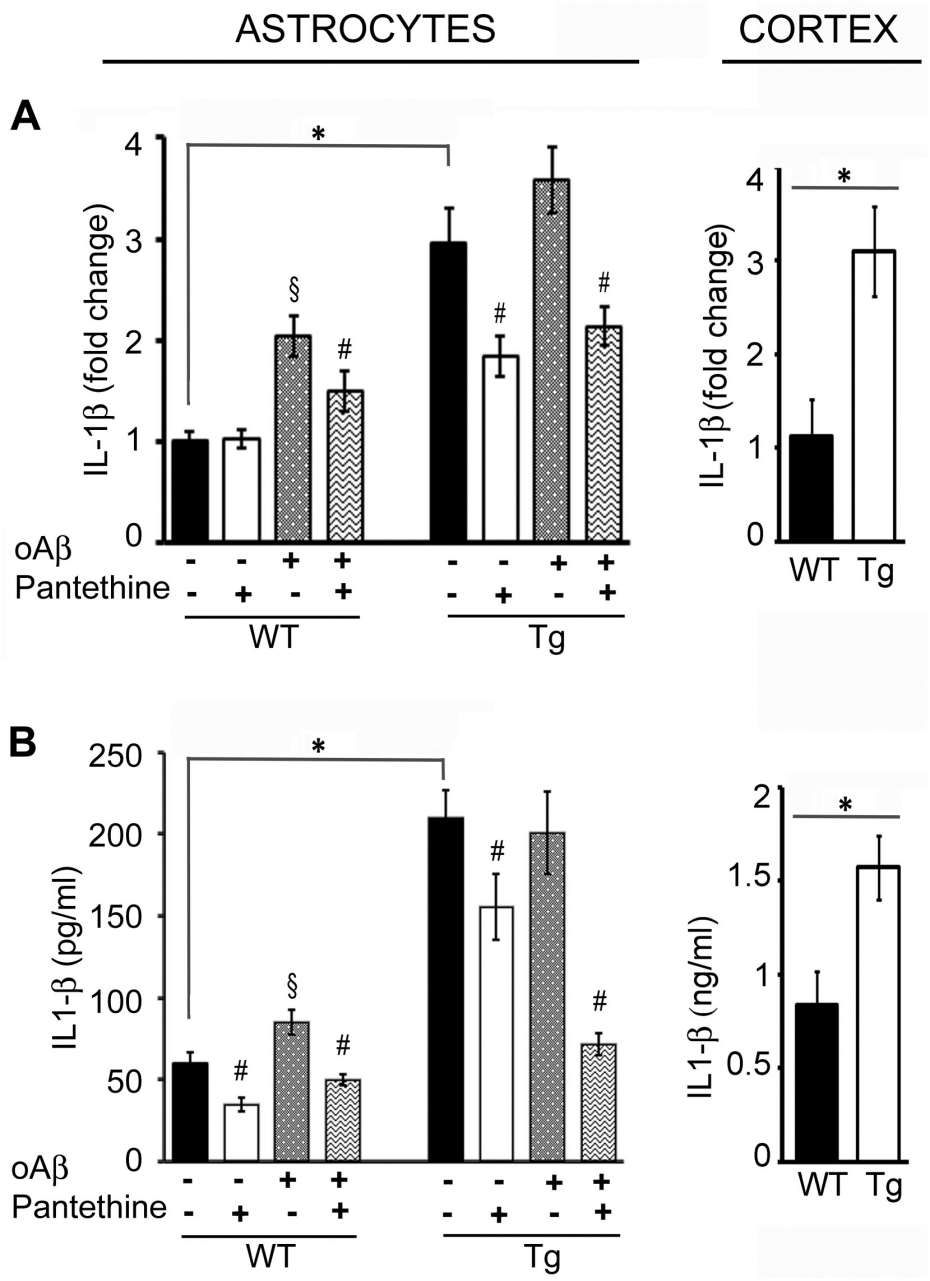
IL-1β expression in WT and Tg astrocytes and cortex. Tg and WT astrocytes were treated or not with pantethine, then exposed or not to oAβ. *IL-1β* mRNA expression was determined on cell extracts; cytokine production was assayed on cell supernatants. In cortex samples collected from mice littermates, mRNA expression and protein production were determined on tissue extracts. (A) qPCR analysis. (B) IL-1β ELISA assays. Results are the mean values ± SD from three independent experiments (n = 3 per group); (*, significant difference between Tg and WT groups; §, significant difference with the corresponding group not exposed to oAβ; #, significant difference with the corresponding group untreated with pantethine; *p*<0.05).

In Tg astrocytes, *IL-1β* mRNA expression showed a 3-fold elevation compared to WT (Fig 4A, left). Exposure to oAβ significantly increased by 2-fold *IL-1β* expression in WT cells, while there were no significant changes in Tg with basal levels already high. The upregulation of *IL-1β* mRNA was found also in the cortex of Tg mice (Fig 4A, right). IL-1β protein production paralleled mRNA expression in the astrocytes as in the cortex (Fig 4B). Pantethine treatment significantly reduced both *IL-1β* mRNA and protein expression in all conditions where it was applied (Fig 4A & 4B).

### Amyloid Precursor Protein (*APP*) and Presenilin 1 (*PS1*) expression

**Fig 5:**
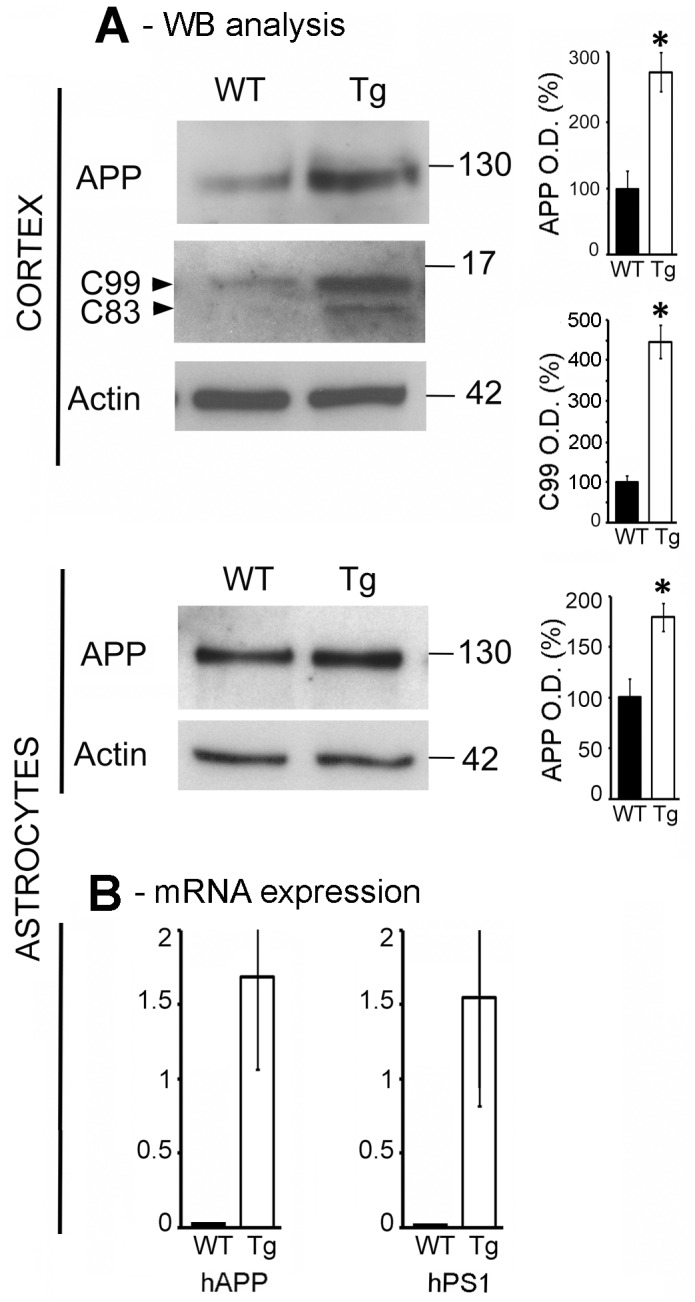
APP is expressed in astrocytes. (A) Western blot analysis of full-length APP, C99 and C83 CTF fragments in Tg and WT cortices (upper panel) and in astrocytes (bottom panel), using a CTF (C-terminal fragment) antibody specifically recognizing the C-terminal end of APP. Graphs on the right represent the mean ± SD of actin-normalized values of the percentage of variation in optical density (O.D.) relative to WT. (B) mRNA expression levels of human APP (*APP*) and presenilin-1 (*PS1*) in Tg and WT astrocytes. Values were obtained from three independent experiments (n = 3 per group) (*, significant difference between Tg and WT groups; *p*<0.05).

We found that the levels of full length APP underwent a significant 2.75-fold increase in Tg cortex compared to WT, as revealed by western blot probed with an antibody (APP-CTF) that specifically recognizes the APP C-terminal end (Fig 5A). Moreover, we observed a significant 4.75-fold increase of APP-derived C99 fragment. Using the same antibody, we found the levels of full length APP increased by 1.75-fold in Tg astrocytes compared with WT (Fig 5B). We confirmed this difference between Tg and WT astrocytes using the 22C11 antibody directed against the N-terminal domain of APP (not shown). We detected expression of both human *APP* and *PS1* mRNAs in Tg astrocytes but not in WT (Fig 5B). These results may suggest higher levels of APP in Tg astrocytes compared to WT. Nevertheless, under our experimental conditions, we failed to detect human APP in the cortex and astrocytes using the human specific 6E10 antibody. In the astrocytes, neither the APP-derived C99 fragment nor the Aß peptide have been detected.

### HIF -1α expression and proteasome activity

**Fig 6:**
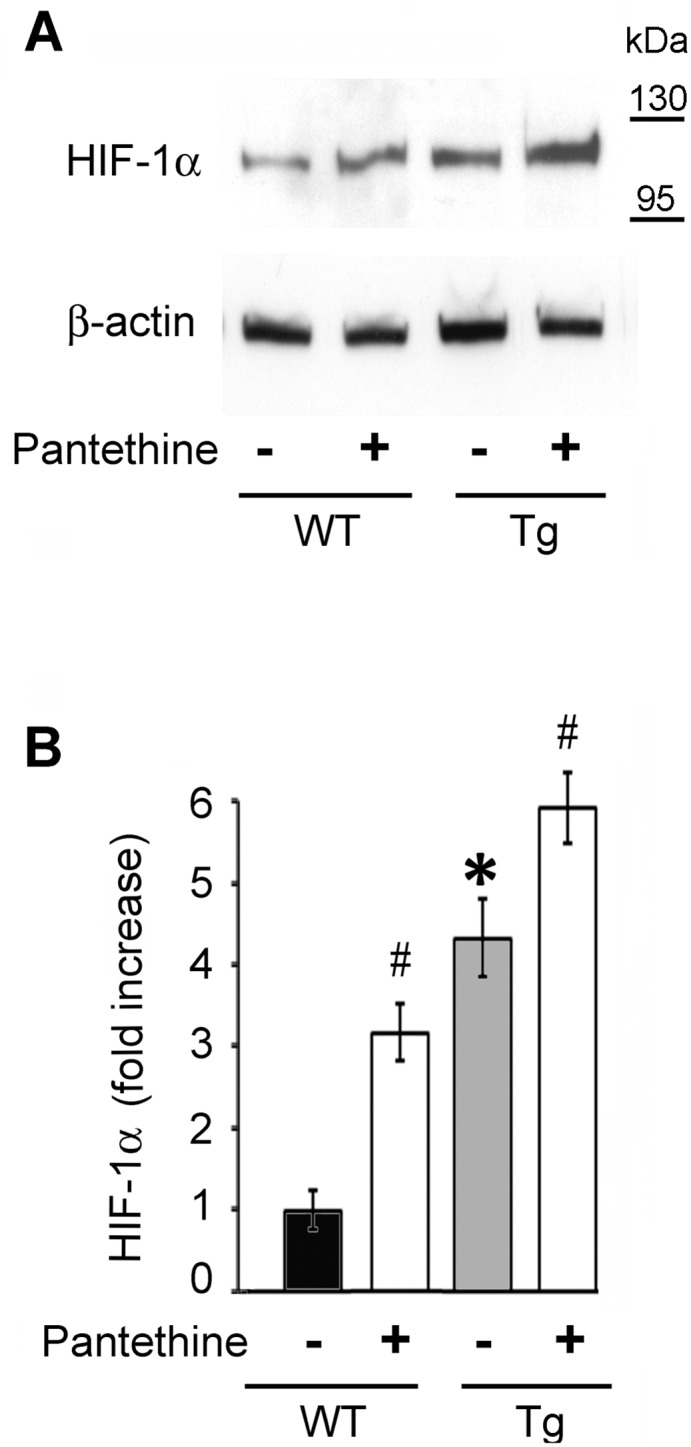
HIF-1α protein levels in Tg and WT astrocytes. (A) Western blot analysis of HIF-1α expression in astrocytes treated or not with pantethine. (B) Densitometric analysis of western blots. Data were normalized to β-actin and are displayed as fold increase relative to untreated WT astrocytes (black diagram). The results represent the mean ± SD from three independent experiments (n = 3 per group) (*, significant difference with the WT control group; #, significant difference with the corresponding group untreated with pantethine; *p*<0.05).

Glial activation and glycolytic changes are known to be reversed by HIF-1α [29]. In normoxic conditions, as those used in our experimental settings, HIF-1α is first hydroxylated by prolyl-hydroxylases (PHDs), then is ubiquitinylated *via* its recognition by the von Hippel-Lindau tumor suppressor protein (pVHL) and finally degraded by the proteasome [29,50]. Such conditions involve the metabolites α-KG, fumarate and succinate (see Fig 2C). We found that untreated Tg astrocytes displayed above 4-fold higher HIF-1α levels than WT astrocytes. Pantethine treatment increased the levels in WT (3-fold) and in Tg (1.5-fold) compared to untreated conditions (Fig 6A and 6B).

**Fig 7:**
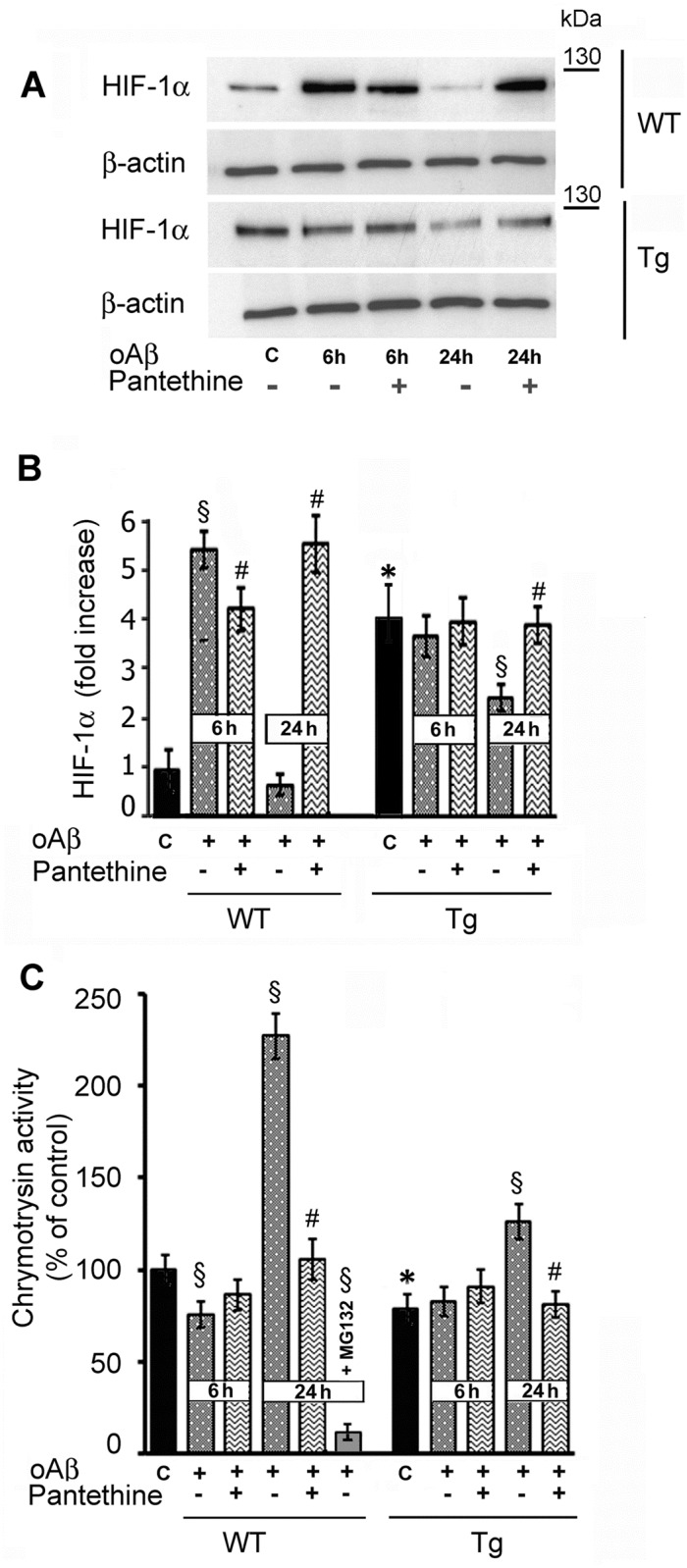
Kinetics of HIF-1α protein expression and proteasome activity in Tg and WT astrocytes following oAβ exposure. (A) Western blot analysis of HIF-1α expression. (B) Densitometry analysis. Data were normalized to β-actin and are displayed as fold increase relative to control untreated WT astrocytes. (C) Proteasomal enzymatic activity assayed using a chymotrypsin substrate. The data are displayed as percent of control untreated WT astrocytes. As a control, enzyme activity was determined in the presence of 0.5 μM of the inhibitor MG132 for 24 h. Bars represent the mean ± SD from three independent experiments (n = 3 per group) (*, significant difference with the corresponding WT control; §, significant difference with the corresponding control; #, significant difference with groups untreated with pantethine; *p*<0.05).

To gain insight into the response of astrocytes to oAβ, we monitored the time course of HIF-1α levels after oAβ exposure (Fig 7A and 7B). At 6 h, a drastic 5-fold increase of HIF-1α levels occurred in WT cells, while no changes were observed in Tg astrocytes, which exhibited high levels under basal conditions. HIF-1α expression was reduced after 24 h in both genotypes, until it was barely detectable in WT astrocytes. Pantethine treatment had little effect on HIF-1α levels at 6 h, while they were increased at 24 h.

As the stability of HIF-1α is mediated mainly by the proteasome, we measured chymotrypsin and trypsin proteasomal activities in astrocytes exposed or not to oAβ. Fig 7C shows that untreated Tg astrocytes exhibited a significant 25% decrease of chymotrypsin activity compared to WT. This reduction likely reflects, at least in part, the increase of HIF-1α levels. After oAβ exposure, the proteasomal activity underwent a moderate decrease at 6 h in WT, while it drastically increased at 24 h, correlating with the fall of HIF-1α levels (Fig 7A and 7B). A comparable, although less marked tendency was also observed in Tg astrocytes. In line with high HIF-1α levels, pantethine treatment resulted in a significant decrease of chymotrypsin proteasomal activity at 24 h. The specificity of the proteosomal assay was confirmed using the MG132 proteasome inhibitor, which reduced chymotrypsin activity by about 90%. Trypsin proteasomal activity was also reduced, but to a lesser extent (data not shown).

## Discussion

In our study we used primary neonatal cultures of cortical astrocytes from Tg and WT mice to investigate alterations of brain metabolism and neuroinflammation associated with AD, and to evaluate the potential of vitamin B5 precursor pantethine to counteract such alterations.

Brain metabolic perturbations have been described in AD patients as well as in transgenic mice [51,52]. We investigated the glycolytic pathway and the TCA cycle. GC/MS characterization yielded distinct metabotypes in Tg compared to WT astrocytes. PK activity, which is involved in the main glycolytic pathway, was decreased while G6PD activity, involved in the alternative pathway (HMS), was stimulated. The latter conserves both the energetic values of glucose and the cell redox status through the production of reducing equivalents [53]. Tg astrocytes exhibited altered levels of some TCA cycle metabolites such as α-KG, succinate and fumarate. These metabolites are substrates and products of α-KGDH and SDH, whose activities were inhibited, in agreement with other reports showing such inhibition in AD [8,54–56]. TCA-cycle intermediate abnormalities have been described in AD patients, in line with decreased activity of key enzymes [9].

The differences that we observed between Tg and WT astrocytes could be linked to the Aβ pathway, since a pattern comparable to Tg was obtained in WT after exposure to oAβ. In agreement with our findings, other studies on primary mouse WT astrocytes have shown that Aβ_1–42_ enhances HMS [29], while Aβ_25–35_ aggregates increased all main glucose metabolic pathways [57]. The relationship between glycolysis and Aβ has been illustrated in human astrocytes, where inhibition of glycolysis renders the normally resistant cells vulnerable to Aβ toxicity [58]. Also, the cortical neurons that are resistant to Aβ toxicity have an enhanced flux of glucose through both the glycolytic pathway and HMS [30].

Pantethine treatment mitigated the metabolic alterations in Tg astrocytes. In the alternative glycolytic pathway, it enhanced G6PD activity, resulting in increased production of the reductant NADPH, which is involved in energy production *via* oxidative phosphorylation (OXPHOS). In the TCA cycle, the treatment alleviated the decline of α-KGDH and SDH activity that occurred in Tg relative to WT, the effect of the treatment being more pronounced in the presence of oAβ. It is noticeable that pantethine treatment ultimately increased ATP production. Similarly to pantethine, known for its anti-cholesterol and hypolipidemic action, DHA treatment of CHO cells was found to mitigate APP-induced impairment of energy metabolism and inflammation by acting on the TCA cycle, cholesterol biosynthesis pathway and fatty acid metabolism [59].

Metabolism alterations may reflect inflammatory conditions. Accordingly, Tg astrocytes displayed a reactive status, as revealed by IL-1β expression and GFAP labeling that contrasted with WT cells. Astrocyte reactivity was associated with expression of the human *APP* and *PS1* mRNAs, suggesting that the neuron-specific Thy1 promoter may also drive transgene mRNA expression in astrocytes. This is in agreement with an early report indicating that Thy1 promoter is functional in long-term cultures of astrocytes [60]. Moreover, activated astrocytes can undergo upregulation of APP levels and processing, leading to an increased production/secretion of Aβ peptides [2]. At this point, the increased levels of full length APP found in Tg astrocytes could account for increased levels of Aß and/or C99, but we failed to observe any difference between genotypes concerning these APP metabolites. In any case, our astrocytes are responsive to changes in Aß, since exposure to exogenous oAβ, increased astrocyte reactivity in both Tg and WT cells, in agreement with previous reports showing that Aβ causes astrocyte activation [26,29].

IL-1β upregulation was not limited to astrocytes as it was found also in the whole cortex of Tg neonatal pups. In this case IL-1ß upregulation was associated with the increase of the C99-APP fragment. The latter has been described as an early neurotoxic metabolite of APP, whose accumulation precedes that of Aβ species and that causes neuroinflammation when overexpressed in the brain [16,17,22,61]. Therefore, cortical astrocytes from newborn pups were likely already in an inflammatory environment. One could expect the Tg astrocytes to return to the WT phenotype once in culture. This was not the case in our study, in agreement with a previous report showing that alterations of GFAP levels and dysfunctions in Aβ clearance remained in 5xFAD astrocytes even after 2–3 weeks in culture [6]. In the 3xTg-AD model, expression of mutated presenilin 1 (PS1_M146V_) was found to alter vesicle dynamics and to reduce peptide secretion in cultured astrocytes devoid of pathologic tissue environment [62]. Altogether, it is possible that perinatal astrocytes exposed to the diseased brain environment may undergo epigenetic changes that persist over the culture period and thus affect sustainably gene expression and function.

Pantethine treatment reduced astrocyte reactivity and IL-1β expression. It can be objected that attenuation of astrocyte reactivity could have adverse effects in AD by accelerating plaque growth, as previously shown [3]. However, this does not apply to our study, which deals with cells isolated during the asymptomatic phase of the disease, prior to detectable Aß accumulation and plaque formation. In the subsequent phases of the disease, *i*.*e*., in Tg mice displaying plaque formation, pantethine treatment led to enhanced GFAP labeling, while IL-1β expression and production in the cortex was inhibited (unpublished results). In comparison, other compounds with anti-inflammatory activity, such as estrogenic compounds or cannabinoid receptor agonists, have been shown to suppress the expression of inflammatory mediators upon Aβ stimulus [63,64].

Astrocyte reactivity and glycolytic changes are known to be reversed by HIF-1α, whose up-regulation is generally associated with neuroprotective mechanisms against various insults [29,30,65]. HIF-1α expression is modulated by TCA metabolites that are involved in the regulation of prolyl hydroxylases (PHDs) [66,67] and proteasomal activity [68]. In our experimental conditions, *i*.*e*., under normoxic conditions, HIF-1α expression was enhanced in Tg compared to WT astrocytes; it was reinforced and more stable across time following exposure to oAβ. In pantethine-pretreated astrocytes, HIF-1α expression was maintained in Tg and strengthened in WT cells.

In summary, we showed that astrocytes generated from asymptomatic 5xFAD pups displayed metabolic features and activated status compared to WT astrocytes. At the same time, protective mechanisms such as sustained HIF-1α expression appeared to emerge. Pantethine treatment mitigated metabolic alteration, dampened inflammation and reinforced HIF-1α expression in Tg astrocytes. Pantethine has the advantage to attenuate pathological mechanisms while it reinforces protective pathways, with no described side-effects. Administered, at the right time during the disease progression, the pleiotropic action of this natural compound could therefore bring improvement in a complex pathological situation such as AD.

## Abbreviations

Aβ: β-amyloid
G6-P: Glucose-6-phosphate
G6PD: Glucose-6-phosphate dehydrogenase
Gly-3P: Glycerol-3-phosphate
HIF-1α: Hypoxia inducible factor-1α
oAβ: Oligomeric Aβ
PEP: Phosphoenoylpyruvate
PK: Pyruvate kinase
rAβ: reverse Aβ
SDH: Succinate dehydrogenase
Sed7P: Sedoheptulose-7-phosphate
α-KGDHc: α-Ketoglutarate dehydrogenase complex
