# Correction: Metabolic changes and inflammation in cultured astrocytes from the 5xFAD mouse model of Alzheimer's disease: Alleviation by pantethine

**DOI:** 10.1371/journal.pone.0194586

**Published:** 2018-03-14

**Authors:** Manuel van Gijsel-Bonnello, Kévin Baranger, Philippe Benech, Santiago Rivera, Michel Khrestchatisky, Max de Reggi, Bouchra Gharib

There is an error in Table 1. The “Pantethine” value in column 5 should be “+”, not “-”.

**Table 1:** Relative abundance of distinct metabolites in Tg and WT astrocytes, treated or not with pantethine. Intracellular levels of different metabolites were determined. The table shows normalized data expressed relative to the abundance of the untreated WT group and are shown as the mean values ± SD from three independent experiments (n = 3 per group); (*, values significantly different between Tg and WT astrocytes; #, values significantly different compared to the corresponding untreated groups; p≤0.05).

Cell type	WT	WT	Tg	Tg
Pantethine	−	+	−	+
Glycolytic pathway				
PEP	1±0.2	1.07±0.19	1.39±0.15*	1.43±0.13
G6-P	1±0.19	0.95±0.17	0.63±0.08*	0.54±0.05
Sed7P	1±0.15	0.91±0.19	0.47±0.09*	0.64±0.08^#^
Gly-3P	1±0.19	0.97±0.16	0.37±0.1*	0.53±0.08^#^
TCA cycle metabolites			
α-KG	1±0.12	0.94±0.13	1.25±0.15*	0.56±0.11^#^
Succinate	1±0.13	0.99±0.12	0.92±0.11	0.72±0.09^#^
Fumarate	1±0.21	0.94±0.19	0.65±0.09*	0.99±0.13^#^
Cis-aconitate	1±0.25	0.94±0.18	1.37±0.3	1.29±0.28
Citrate	1±0.25	0.86±0.21	1.24±0.29	1.26±0.22
Nucleotides				
ATP	1±0.08	0.97±0.12	1.01±0.13	1.44±0.14^#^
AMP	1±0.07	0.90±0.19	0.80±0.11*	1.07±0.15
CMP	1±0.15	0.96±0.19	0.60±0.11*	1.00±0.22^#^
CTP	1±0.12	0.98±0.15	1.03±0.11	1.16±0.13
GDP	1±0.15	0.95±0.13	0.91±0.11	1.03±0.16
ADP	1±0.18	0.98±0.16	1.10±0.12	1.19±0.16
